# Design, Synthesis, Experimental and Theoretical Characterization of a New Multitarget 2-Thienyl-*N*-Acylhydrazone Derivative

**DOI:** 10.3390/ph11040119

**Published:** 2018-11-01

**Authors:** Isadora T. S. Bastos, Pedro de Sena M. Pinheiro, Fanny N. Costa, Miguel D. Rocha, Carlos Mauricio R. Sant’Anna, Delson Braz, Everton T. Souza, Marco A. Martins, Eliezer J. Barreiro, Fabio F. Ferreira, Regina C. Barroso, Carlos A. M. Fraga

**Affiliations:** 1 LabFisMed, State University of Rio de Janeiro, Physics Institute, Rio de Janeiro 20550-900, RJ, Brazil; isadoratairinne@gmail.com; 2 Laboratório de Avaliação e Síntese de Substâncias Bioativas (LASSBio), Instituto de Ciências Biomédicas, Universidade Federal do Rio de Janeiro, P.O. Box 68023, Rio de Janeiro 21941-902, RJ, Brazil; pedro_senamp@hotmail.com (P.d.S.M.P.); miguelquimic@gmail.com (M.D.R.); santanna@ufrrj.br (C.M.R.S.); ejbarreiro@ccsdecania.ufrj.br (E.J.B.); 3 Programa de Pós-Graduação em Farmacologia e Química Medicinal, Instituto de Ciências Biomédicas, Universidade Federal do Rio de Janeiro, Rio de Janeiro 21941-902, RJ, Brazil; evertontenoriosouza@gmail.com (E.T.S.); mamartins56@me.com (M.A.M.); 4 Center for Natural and Human Sciences, Federal University of ABC, Santo André 09210-580, SP, Brazil; fannycosta@yahoo.com.br (F.N.C.); ffurlanf@gmail.com (F.F.F.); 5 Departamento de Química Fundamental, Instituto de Química, Universidade Federal Rural do Rio de Janeiro, Seropédica 23970-000, RJ, Brazil; 6 Laboratory of Nuclear Instrumentation/COPPE, Federal University of Rio de Janeiro, Rio de Janeiro 21949-900, RJ, Brazil; delson@nuclear.ufrj.br; 7 Laboratório de Inflamação Instituto Oswaldo Cruz, Fundação Oswaldo Cruz, Rio de Janeiro 21045-900, RJ, Brazil

**Keywords:** *N*-acylhydrazone, PDE4 inhibitor, adenosine A_2A_ receptor, X-ray powder diffractometry, crystal structure determination, chalcogen bond, sigma-hole

## Abstract

Pulmonary arterial hypertension (PAH) is a chronic cardiovascular disease that displays inflammatory components, which contributes to the difficulty of adequate treatment with the available therapeutic arsenal. In this context, the *N*-acylhydrazone derivative LASSBio-1359 was previously described as a multitarget drug candidate able to revert the events associated with the progression of PAH in animal models. However, in spite of having a dual profile as PDE4 inhibitor and adenosine A_2A_ receptor agonist, LASSBio-1359 does not present balanced potencies in the modulation of these two targets, which difficult its therapeutic use. In this paper, we describe the design concept of LASSBio-1835, a novel structural analogue of LASSBio-1359, planned by exploiting ring bioisosterism. Using X-ray powder diffraction, calorimetric techniques, and molecular modeling, we clearly indicate the presence of a preferred synperiplanar conformation at the amide function, which is fixed by an intramolecular 1,5-N∙∙∙S σ-hole intramolecular interaction. Moreover, the evaluation of LASSBio-1835 (**4**) as a PDE4 inhibitor and as an A_2A_ agonist confirms it presents a more balanced dual profile, being considered a promising prototype for the treatment of PAH.

## 1. Introduction

The conformational restriction of a flexible ligand can be used to fix a certain conformation capable of responding to molecular recognition by a particular receptor site with selectivity. The rational control of the conformation in small molecules is fundamental for structure- and ligand-based molecular design and, for this reason, several strategies to influence conformational preferences have been widely applied for these purposes [1,2,3].

There are several examples in the literature that describe the use of different structural modifications to promote changes in the conformational behavior and in the bioactivity profile of bioactive compounds as reported previously for the *N*-acylhydrazone derivative LASSBio-294 and the *N*-methyl-*N*-acylhydrazone derivative LASSBio-785 [4,5]. For these compounds, the X-ray crystallography analysis, molecular modeling, and ultraviolet spectroscopy elucidated the bioactive conformations, revealing that the methylation of the *N*-acylhydrazone leads to an impressive conformational change that might be responsible for the different pharmacological properties of these two derivatives [4,5].

LASSBio-294, in solid state, presents a planar conformation (amide hydrogen antiperiplanar to the carbonyl oxygen at the *N*-acylhydrazone moiety) and LASSBio-785 tends to turn 180° into amide bond, generating a different folded conformation (amide hydrogen synperiplanar to the carbonyl oxygen) [4]. Moreover, we have described earlier the participation of a new type of σ-hole intramolecular interaction between the sulfur atoms at the thiophene ring and the nitrogen atoms at the imine subunit of these *N*-acylhydrazone derivatives, which contribute to the stabilization of a particular molecular conformation and has an influence on its molecular properties [6]. This attractive interaction is based on the presence of positive electrostatic potential regions on the sides of the aromatic sulfur atom, that is, the *σ*-holes regions that are available for intramolecular interaction with Lewis basis, such as nitrogen and oxygen atoms [7,8].

In this context, we described the development of two new thienyl-*N*-acylhydrazone derivatives, LASSBio-1834 (**3**) and LASSBio-1835 (**4**) [9], which were planned as multitarget vasodilator and anti-inflammatory prototypes, presenting an unprecedented structural pattern and bioactivity profile capable of being exploited in the treatment of pulmonary arterial hypertension [10].

**Figure 1:**
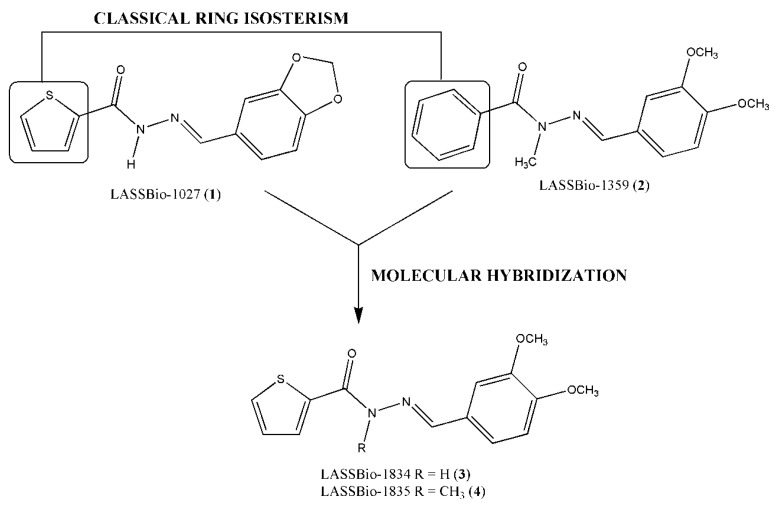
Design concept of *N*-acylhydrazone derivatives LASSBio-1834 (**3**) and LASSBio-1835 (**4**).

The new *N*-acylhydrazone derivative LASSBio-1834 (**3**) and its *N*-methyl-*N*-acylhydrazone analogue LASSBio-1835 (**4**) were structurally planned by applying the strategies of molecular hybridization [11] and ring bioisosterism [12,13] (Figure 1), having as starting compounds LASSBio-1027 (**1**) and LASSBio-1359 (**2**), which were, respectively, characterized as adenosine A_2A_ receptor agonist [14] and as selective phosphodiesterase 4 inhibitor [15]. The structure of the planned compounds combined the 3,4-dimethoxyphenyl group of (**2**), which is a well-known pharmacophore for PDE4 inhibition, with the thienyl-*N*-acylhydrazone framework of (**1**), varying the methylation pattern at the amide nitrogen of the NAH subunit.

The goal of the present study is to investigate the anticipated multitarget profile of NAH derivatives LASSBio-1834 (**3**) and LASSBio-1835 (**4**) through in vitro assays, determine the crystal structure of these compounds in solid state, since their conformations and relative configuration could be associated to their bioactivity profiles, and, in addition, to perform theoretical studies by means of computational methods to corroborate our results.

## 2. Results and Discussion

### 2.1. Synthesis of Thienyl-N-Acylhydrazone Derivatives *(**3**)* and *(**4**)*

**Scheme 1:**
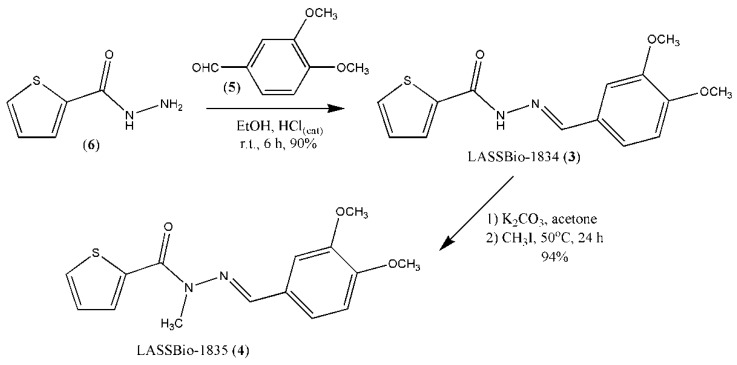
Synthesis of *N*-acylhydrazone derivatives (**3**) and (**4**).

The title NAH compounds were prepared through a classical synthetic methodology that exploited the acid-catalyzed condensation of 2-thienylcarbohydrazide (**6**) with 3,4-dimethoxybenzaldehyde (**5**), both obtained from commercial sources (Scheme 1) [4,15]. In this condition, (3,4-dimethoxybenzylidene)-2-thienylhydrazide LASSBio-1834 (**3**) was obtained in 90% yield; next, it was converted to the corresponding *N*-methyl-*N*-acylhydrazone derivative (LASSBio-1835 (**4**)) after regioselective alkylation of the amide nitrogen, a resultant from treatment with methyl iodide under basic conditions (Scheme 1) [4,15].

As previously described by our research group, the condensation between hydrazides and aldehydes preferentially produces *N*-acylhydrazones with a relative (*E*) configuration [4,15,16,17]. In agreement with this behavior, the analysis of ^1^H and ^13^C NMR spectra of 2-thienyl-*N*-acylhydrazones (**3**) and (**4**) indicated they were obtained as a single diastereoisomer, which X-ray diffraction studies confirmed to present the relative configuration (*E*), as will be discussed next.

### 2.2. In Vitro Pharmacological Studies

Evaluation of the potential inhibitory activity of the 2-thienyl-*N*-acylhydrazone derivatives LASSBio-1834 (**3**) and LASSBio-1835 (**4**) on human recombinant isoforms of PDE4 (PDE4A, 4B, 4C and 4D) provided evidence of a selective profile for PDE4A1A and PDE4B1. When tested at screening concentration of 10 µM LASSBio-1834 (**3**) inhibited PDE4A1A in 53%, PDE4B1 in 48%, PDE4C in 12%, and PDE4D3 in 19%. On the other hand, LASSBio-1835 (**4**) inhibited the catalytic activity of PDE4A1A in 78%, PDE4B1 in 44%, PDE4C in 22%, and PDE4D3 in 40%. The PDE inhibition data are summarized in Table 1.

The most effective compound LASSBio-1835 (**4**) had its concentration inhibitory response curve determined for PDE4A1A, exhibiting an IC_50_ of 1.08 µM. As previously described by Kummerle et al. [15] the *N*-methylation of *N*-acylhydrazone derivatives potentialize its action as PDE4 inhibitors for stabilizing the bioactive conformation, which is related to a synperiplanar conformation of the methyl group with respect to the carbonyl oxygen of the NAH subunit. The structural and in silico studies described herein clearly corroborate this evidence.

The best PDE4 inhibitor LASSBio-1835 (**4**) was submitted to binding assays to the human recombinant adenosine A_2A_ receptors [15], by using [^3^H]-CGS21680 as a selective agonist radioligand for this receptor subtype. The results indicated that compound (**4**) show a moderate affinity for A_2A_ receptors with an IC_50_ of 1.8 µM (Ki = 1.5 µM).

The in vitro pharmacological studies clearly demonstrate the multi-target profile of LASSBio-1835 (**4**), in which the selective modulation of the two targets, PDE4A1 and A_2A_ receptors, represent a novel strategy for the treatment of pulmonary arterial hypertension.

### 2.3. Structure-Property Evaluation of Thienyl-N-Acylhydrazone Derivatives *(**3**)* and *(**4**)*

**Figure 2:**
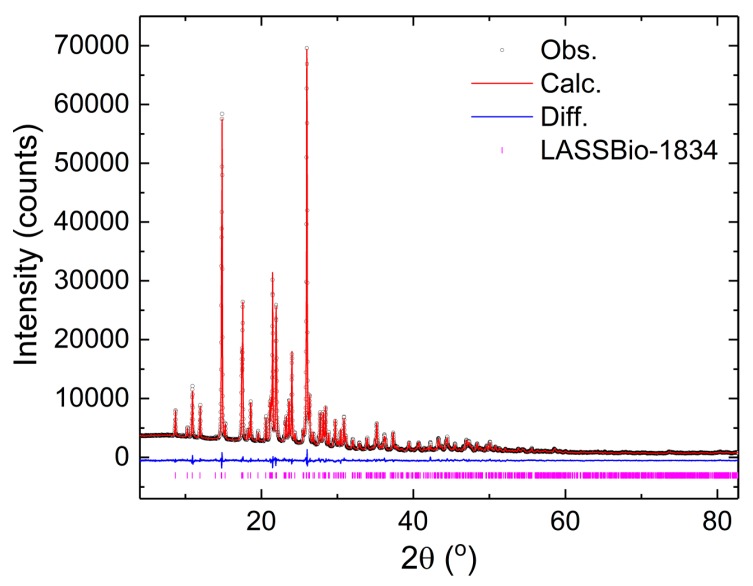
Plot from the final Rietveld refinement of LASSBio-1834 (**3**). Black circles represent observed data; the red line indicates the calculated pattern and the blue line at the bottom represents the difference between the observed and calculated patterns. Magenta vertical bars indicate the Bragg reflections.

**Figure 3:**
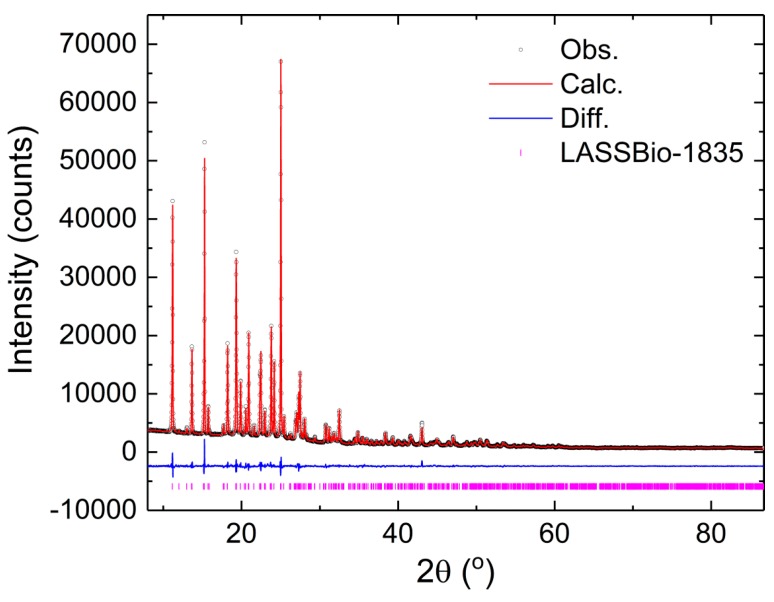
Plot from the final Rietveld refinement of LASSBio-1835 (**4**). Black circles represent observed data; the red line indicates the calculated pattern and the blue line at the bottom represents the difference between the observed and calculated patterns. Magenta vertical bars indicate the Bragg reflections.

After the Rietveld refinements, the values found for the unit cell parameters for both (**3**) and (**4**) were, respectively: *a* = 10.18564(17) Å, *b* = 16.2171(2) Å, *c* = 8.34244(15) Å, *β* = 90.3476(10)° and V = 1377.99(4) Å^3^ and *a* = 13.6197(2) Å, *b* = 9.73633(13) Å, *c* = 11.26776(17) Å, *β* = 90.4210(10)° and V = 1494.13(4) Å^3^. The goodness-of-fit indicators and *R*-factors were, respectively: (**3**): χ^2^ = 1.683, *R_Bragg_* = 1.757%, *R_wp_* = 3.575% and *R_exp_* = 2.124% and (**4**) χ^2^ = 1.548, *R_Bragg_* = 1.635%, *R_wp_* = 3.495% and *R_exp_* = 2.257%. Figure 2 and Figure 3 display the final Rietveld plots for (**3**) and (**4**), respectively.

**Figure 4:**
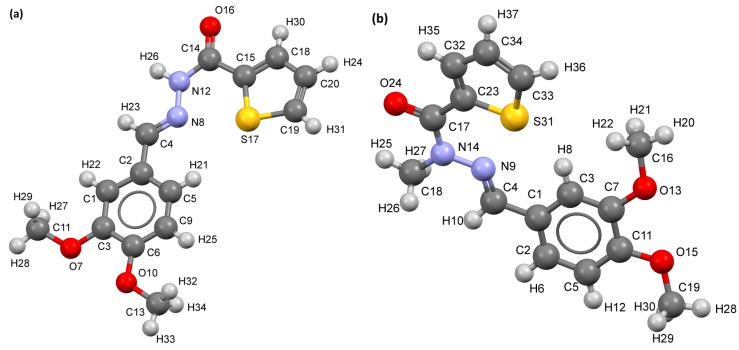
Molecular structure of LASSBio-1834 (**3**) (**a**) and LASSBio-1835 (**4**) (**b**) compound indicating all atoms as spheres.

The crystal structures of LASSBio-1834 (Figure 3) and LASSBio-1835 (Figure 4) adopted the relative configuration *E* in relation to the imine double bond (Figure 4). Also, the preference for a synperiplanar conformer in the amide in solid-phase was also evidenced (Figure 4). This observation was surprising for LASSBio-1834 (Figure 3), since it is expected to find an antiperiplanar conformation in the amide of NAH derivatives without substitution at the amide nitrogen [4,5]. Crystal data, as well the details of the structures, are shown in Table S1 (see Supplementary Materials).

The formation of crystalline aggregates of LASSBio-1834 (Figure 3) molecules in the solid phase is shown in Figure S1a (see Supplementary Materials), with the intermolecular hydrogen bond interactions evidenced by the cyan dashed lines. The molecules are held together by hydrogen bond interactions between the carbonyl oxygens and the amide hydrogens of the *N*-acylhydrazone function. Figure S1b (see Supplementary Materials) displays the crystalline aggregate of LASSBio-1835 (Figure 4) molecules in solid phase. Possible hydrogen bond interactions can be observed by the cyan dashed lines, in which the intermolecular interactions between the methoxy oxygen atoms and the methyl hydrogens atoms are responsible for maintaining the molecules together in the crystalline state.

It is worth mentioning that both compounds present similar conformations in solid state, demonstrating that there are possibly other ways of inducing this type of conformation, in which not only the *N*-methylation of *N*-acylhydrazone derivatives can cause a conformational change at the amide function from an antiperiplanar conformation to a synperiplanar conformation [4]. On the other hand, from the ^1^H NMR data in DMSO-*d*_6_ it is possible to observe a mixture of conformers for LASSBio-1834 (Figure 3) indicating that there is an equilibrium between the antiperiplanar and synperiplanar conformations (Figure S4, see Supplementary Materials); however, the same behavior was not observed for LASSBio-1835 (Figure 4) (Figure S7, see Supplementary Materials). Taken together, these results indicate that the possible reason for LASSBio-1834 (Figure 3) being less potent to inhibit PDE4A1A in relation to LASSBio-1835 (Figure 4) is that the bioactive conformation is not fully stabilized in the solution. The substitution at the amide nitrogen of the NAH subunit in the structure of LASSBio-1835 (Figure 4) helped the conformational restriction of this compound into the bioactive conformation.

The folded conformation found in the solid state for these compounds is possibly being stabilized by 1,5-N∙∙∙S sigma-hole (σ-hole) intramolecular interactions in both systems, which is corroborated by the distance between the imine nitrogen and sulfur atoms in (**3**) (D_N∙∙∙S_ = 2.695 Å) and (**4**) (D_N∙∙∙S_ = 2.693 Å) being less than the sum of their van der Waals radii (Figure 4). Moreover, the dihedral angle formed between the thiophene ring and the imine moiety in (**3**) (ϕ_S17C15N12N8_ = −3.86°) and (**4**) (ϕ_S31C23N14N9_ = −6.42°) also corroborate the presence of 1,5-N∙∙∙S σ-hole interactions in both compounds (Figure 4). Aiming to understand the σ-hole interactions between the imine nitrogen and sulfur atoms in LASSBio-1834 (**3**) and LASSBio-1835 (**4**), we performed theoretical evaluations to analyze these interactions.

**Figure 5:**
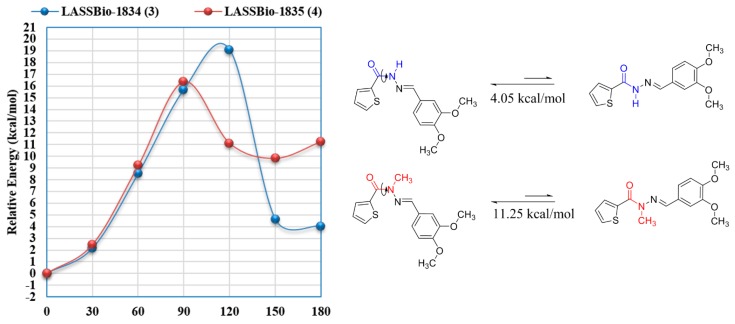
CAM-B3LYP/6-31G(d) potential energy surfaces of the O=C-N-X dihedral angle for LASSBio-1834 (**3**) and LASSBio-1835 (**4**), obtained with the SPARTAN’16 software (Wavefunction, Inc.).

**Figure 6:**
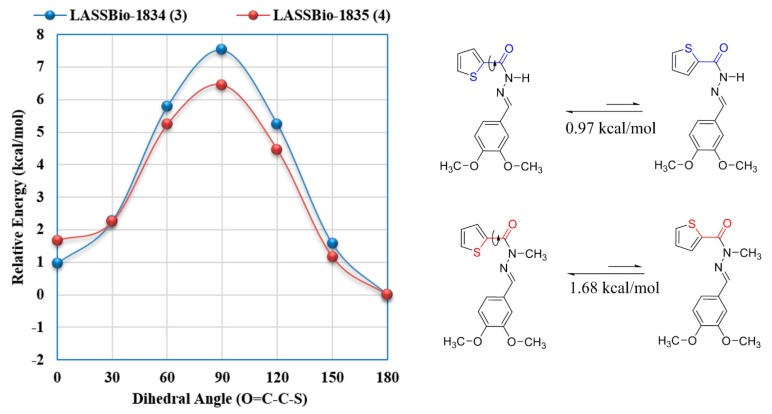
CAM-B3LYP/6-31G(d) potential energy surfaces of the O=C-C-S dihedral angle for LASSBio-1834 (**3**) and LASSBio-1835 (**4**), obtained with the SPARTAN’16 software (Wavefunction, Inc., Irvine, CA, USA).

In order to understand the molecular reasons responsible for the crystallographic conformations of LASSBio-1834 (**3**) and LASSBio-1835 (**4**), we calculated the PES scans of the dihedral angles highlighted in Figure 5 and Figure 6 in gas phase. First, we analyzed the amide bond conformation, changing the O=C-N-X (X = H (**3**) or CH_3_ (**4**)) dihedral angle from 0° to 180°. According to the results, both molecules are significantly stable in the synperiplanar conformation, with an energy difference between the synperiplanar and antiperiplanar conformations of 4.05 kcal∙mol^−1^ for LASSBio-1834 (**3**) and 11.25 kcal∙mol^−1^ for LASSBio-1835 (**4**), in accordance with the experimental data (Figure 5). The anti-syn energy difference is significantly higher for LASSBio-1835 (**4**), which is probably related to the steric effect promoted by the presence of the methyl group. The Boltzmann distribution analysis indicated that the lower energy conformers represent 97.3% and 98.5% of the analyzed conformers of LASSBio-1834 (**3**) and LASSBio-1835 (**4**), respectively.

We also analyzed by the same method the S-C-C=O torsion angle, since it would be possible to have two different σ-hole intramolecular interactions occur in both molecules (1,4-O∙∙∙S and 1,5-N∙∙∙S) (Figure 6). It can be seen that the molecules are more stable with an S-C-C=O dihedral angle of 180° by 0.97 and 1.68 kcal∙mol^−1^ for LASSBio-1834 (**3**) and LASSBio-1835 (**4**), respectively, in accordance with the experimental data (Figure 6). The anti-conformers are more stable, most likely because the 1,5-N∙∙∙S σ-hole interaction is stronger than the 1,4-O∙∙∙S σ-hole interaction, since the 1,5 relationship is mimicking a six-membered ring, which is more stable than a five-membered ring, represented by the 1,4 relationship. The Boltzmann distribution analyzes indicated that the anti-conformers in relation to the S-C-C=O torsion angle represent 77.7% and 82.0% of the analyzed conformers for LASSBio-1834 (**3**) and LASSBio-1835 (**4**), respectively.

**Figure 7:**
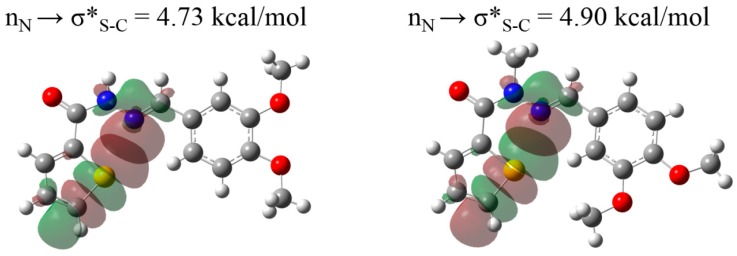
Natural Bond Orbital (NBO) analysis evaluation of the n_N_ → σ*_S-C_ interaction using the crystallographic coordinates of LASSBio-1834 (**3**) and LASSBio-1835 (**4**). The NBO analyses were performed using the CAM-B3LYP/6-311G(d) level of theory using the GAUSSIAN’09 software.

According to the crystallographic structures of LASSBio-1834 (**3**) and LASSBio-1835 (**4**), the imine nitrogen and sulfur atoms were separated by 2.695 and 2.693 Å, respectively; less than the sum of their van der Waals radii, which is another indication of the 1,5-N∙∙∙S interaction [7]. In this way, we analyzed the crystallographic structures in terms of a Natural Bond Orbital (NBO) analysis, using the CAM-B3LYP/6-311G(d) level of theory to specifically evaluate the 1,5-N∙∙∙S σ-hole intramolecular interactions. The NBO analysis indicated a direct orbital interaction between the imine nitrogen electron lone pair (n_N_) and one of the σ* orbital of the sulfur-carbon bonds (σ*_S-C_) in both systems (Figure 7). As expected by the similar distance values, the n_N_ → σ*_S-C_ interaction had a similar strength in both systems, being 4.73 kcal∙mol^−1^ in LASSBio-1834 (**3**) and 4.90 kcal∙mol^−1^ in LASSBio-1835 (**4**) (Figure 7). The small increase in the strength of the n_N_ → σ*_S-C_ interaction for LASSBio-1835 (**4**) is probably related to the methyl group presence in its structure, which can contribute to the increase of the imine nitrogen electron density. The theoretical results are in good agreement with the experimental data, showing that the unusual conformation of the analyzed compounds was mainly caused by a 1,5-N∙∙∙S σ-hole intramolecular interaction.

The SEM images of LASSBio-1834 (**3**) and LASSBio-1835 (**4**) compounds were obtained in order to analyze the habit of the formed crystals. This allows us to give a greater characterization of these compounds.

**Figure 8:**
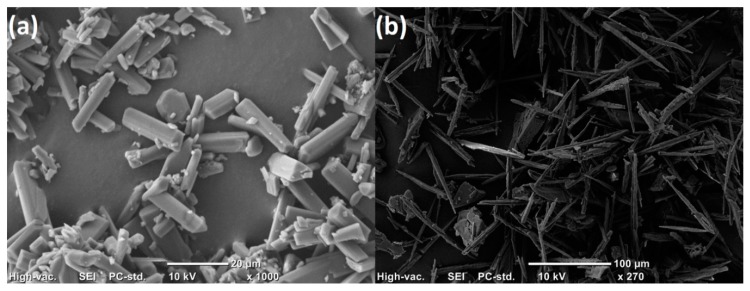
Scanning electron microscopy (SEM) image of LASSBio-1834 (**3**) crystals exhibiting plates shapes and relatively smooth surfaces (**a**) and LASSBio-1835 (**4**) crystals exhibiting needle-like shapes (**b**).

Figure 8a shows the acquired image of some LASSBio-1834 (**3**) crystals, exhibiting a plate’s shapes and relatively smooth surfaces. In general, the crystals have a thicknesses up to 5 mm and longer lengths. LASSBio-1835 (**4**) crystals, Figure 8b, show a needle-like shape. These results are interesting because they have completely different shapes, which can be in part responsible for the differences in water solubility of these compounds, i.e., 20.4 µM and 2.8 µM, respectively, for LASSBio-1834 (**3**) and LASSBio-1835 (**4**). This physicochemical parameter is well known as being important in the discovery and developmental stages during the search for new drug candidates, due to its capability to interfere with the performance in both the in vitro and in vivo pharmacological assays [18].

Moreover, the DSC scans of LASSBio-1834 (**3**) and LASSBio-1835 (**4**) (Figure S2, see Supplementary Materials) show a well-defined melting behavior with a sharp minimum, which easily allowed us to determine the corresponding melting temperature (440.40 K and 415.41 K, respectively). Then, we could infer the enthalpy of fusion for both compounds as being ΔH = 40.11 kJ∙mol^−1^ for LASSBio-1834 (**3**) and ΔH = 32.07 kJ∙mol^−1^ for LASSBio-1835 (**4**).

## 3. Experimental Section

### 3.1. Chemistry

The progress of all reactions was monitored by thin layer chromatography (TLC), which was performed on aluminum sheets, pre-coated with silica gel 60 (HF-254; Merck, Darmstadt, Germany) to a thickness of 0.25-mm. The recorded chromatograms were acquired under ultraviolet light (254–366 nm). Reagents and solvents were purchased from commercial suppliers and used as received. Melting points were determined with a Quimis 340 apparatus. ^1^H-NMR spectra were recorded in deuterated chloroform (CDCl_3_) or dimethylsulfoxide (DMSO-*d*_6_) with a Bruker DPX-200 spectrometer at 200 MHz. ^13^C-NMR spectra were recorded with the same spectrometer at 50 MHz with the same solvents.

Analytical HPLC was performed using a Shimadzu LC-20AD with a Kromasil 100-5 C18 (4.6 mm × 250 mm) and a Shimadzu SPD-M20A detector (diode array) at 254 nm wavelength. The solvent system used for HPLC analyses was acetonitrile:water (60:40). The isocratic HPLC mode was used, and the flow rate was 1.0 mL∙min^−1^. The purity of the compounds was found to be greater than 95%. The HPLC solvent (acetonitrile) was purchased from Sigma-Aldrich (St. Louis, MO, USA). Water used in the preparations has been previously purified and filtered using a Milli-Q system (Millipore, St Quentin-en-Yvelines, France). The high resolution mass spectrometric analyzes were carried out by ionization in positive mode, in an UltrOTOF Mass Spectrometer (Bruker Daltonics, Billerica, MS, USA) at LabMass-IPPN (UFRJ). High resolution mass spectra (HRMS) were processed in the Bruker Compass DataAnalysis 4.0 software.

To obtain a solution of 3,4-dimetoxybenzaldeyde (0.715 g, 4.30 mmol) in absolute EtOH (5 mL) containing one drop of 37% hydrochloric acid, was added to a solution of 2-thyenylhydrazide (0.600 g, 4.22 mmol), commercially obtained, in absolute EtOH (5 mL). The mixture was stirred at room temperature for 6 h until extensive precipitation was visualized. Afterwards, the solvent was partially concentrated at reduced pressure and the resulting mixture was poured into cold water. After neutralization with 10% aqueous sodium bicarbonate solution, the precipitate formed was filtered out and dried under vacuum, producing the desired (3,4-dimethoxybenzylidene)-2-thienylhydrazide LASSBio-1834 (**3**) as a white solid in 90% yield, mp. 452.15–455.15 K. ^1^H-NMR (200 MHz, CDCl_3_, δ (ppm)): 11.00 (*s*, 1H, -CONH-), 8.24 (*s*, 1H, thienyl-H_5_), 8.00 (*s*, 1H, N=CH), 7.68 (*d*, 1H, thienyl-H_3_, *J* = 3.9 Hz), 7.51 (*s*, 1H, phenyl-H_2_), 7.17–7.21 (*m*, 2H, thienyl-H_4_ e phenyl-H_6_), 6.89 (*d*, 1H, phenyl-H_5_, *J* = 8.0 Hz), 3.92 (*s*, 3H, -OCH_3_) and 3.97 (*s*, 3H, -OCH_3_). ^13^C-NMR (50 MHz, CDCl_3_, δ (ppm)): 163.3, 151.2, 149.5, 145.0, 135.6, 134.5, 132.8, 127.1, 126.6, 122.4, 110.9, 109.2, 56.1 and 56.0. Purity (HPLC): 97.7% (λ 328 nm, RT 4.13 min). HRMS (ESI, *m*/*z*): calculated for [M + Na] C_14_H_14_N_2_O_3_SNa, 313.0617, found 313.0616.

The (3,4-dimethoxybenzylidene)-2-thienylhydrazide LASSBio-1834 (**3**) (0.5 g, 1.72 mmol) and potassium carbonate (0.715 g, 5.2 mmol) were suspended in 10 mL of acetone. The suspension was thoroughly mixed under vigorous stirring for 5 min, and then, methyl iodide (0.730 g, 5.2 mmol) was added. The reaction was heated at 323.15 K and maintained under stirring for 24 h. Next, the solvent was partially concentrated at reduced pressure, and the residual solid was suspended in 5 mL of water and then poured into cold water. The precipitate formed was filtered out, washed with cold water, dried under vacuum, to generate the desired (3,4-dimethoxybenzylidene)-*N*-methyl-2-thienylhydrazide LASSBio-1835 (**4**), with 94% yield, as a white solid, mp. 421.15–424.15 K. ^1^H-NMR (200 MHz, CDCl_3_, δ (ppm)): 8.18 (*dd*, 1H, thienyl-H_5_, *J* = 3.8 and 1.2 Hz), 7.79 (*s*, 1H, =CH-), 7.65 (*d*, 1H, phenyl-H_2_, *J* = 1.8 Hz), 7.61 (*dd*, 1H, thienyl-H_3_, *J* = 5.1 and 1.2 Hz), 7.24 (*dd*, 1H, phenyl-H_6_, *J* = 8.2 and 1.8 Hz), 7.12 (*dd*, 1H, thienyl-H_4_, *J* = 5.1 and 3.8 Hz), 6.92 (*d*, 1H, phenyl-H_5_, *J* = 8.2 Hz), 3.98 (*s*, 3H, -OCH_3_), 3.93 (*s*, 3H, -OCH_3_) and 3.55 (*s*, 3H, -NCH_3_-). ^13^C-NMR (50 MHz, CDCl_3_, δ (ppm)): 162.4, 150.9, 149.6, 140.0, 136.3, 134.2, 133.0, 127.8, 126.3, 122.6, 111.0, 109.6, 56.1 and 28.8. Purity (HPLC): 99.5% (λ 330 nm, RT 7.33 min). HRMS (ESI, *m*/*z*): calculated for [M + Na] C_15_H_16_N_2_O_3_SNa, 327.0774, found 327.0778.

### 3.2. In vitro Pharmacological Studies

PDE4 inhibitory activity (PDE4A, 4B, 4C, and 4D) was measured by employing an IMAP^TM^ FP PDE Evaluation Assay Kit (Molecular Devices, Sunnyvale, CA, USA). Enzymatic reactions were carried out at room temperature in a 96-well black plate by co-incubating 25 μL of 200 nM FAM-cAMP (R7513), 5 μL of putative inhibitory compounds, and 20 μL of the PDE4 isoform dissolved in assay buffer (R7364) for 1 h. All enzymes were obtained from human recombinant sources (MDS PHARMA), whereas the other reagents were purchased from Molecular Devices. Fluorescence polarization intensity was measured at 485 nm excitation and 520 nm emission using a microplate reader, SpectraMax M5 (Molecular Devices, Sunnyvale, CA, USA). PDE4 inhibitors were dissolved in dimethylsulfoxide (DMSO) at a final concentration of 0.1%. At this condition, the vehicle had no significant effect on PDE4 activity. The concentration of the prototypes that produced 50% inhibition of substrate hydrolysis (IC_50_) was calculated by non-linear regression analysis from concentration response curves. LASSBio-1834 and LASSBio-1835 were tested at 10 µM concerning screening conditions. The latter compound was also tested in a range of concentrations aiming at the IC_50_ value.

The binding assays to the A_2A_ adenosine receptors were made in EUROFINS-CEREP France (www.cerep.fr) under the following study number: 100016552, using the protocol previously published by Luthin and Linden (1995) [15]. The human recombinant A_2A_ receptors were expressed in HEK 293 cells and binding was performed with [^3^H]-CGS21680 a selective agonist radioligand for this receptor subtype. The analysis was performed using software developed at Cerep (Hill software) and GraphPad Prism^®^ 5.0 (GraphPad Software, Inc., San Diego, CA, USA).

### 3.3. Differential Scanning Calorimetry

The Differential Scanning Calorimetry (DSC) curves for both compounds were obtained in a differential scanning calorimeter, model DSC Q200, from TA Instruments, with heat flux using a temperature range from 293 K to 523 K and a heating rate of 10 K∙min^−1^. Small portions of the samples, with a mass of 4.27 mg for LASSBio-1834 and 4.29 mg for LASSBio-1835, were transferred to an aluminum crucible, previously weighed. The resulting thermograms were analyzed with the TA Universal Analysis software revealing that the samples’ purities were 99.6% and 97.2%, respectively.

### 3.4. X-ray Powder Diffraction

The LASSBio-1834 (**3**) and LASSBio-1835 (**4**) samples were gently hand-ground in a pestle and mortar and the X-ray powder diffraction (XRPD) were recorded at room temperature on a STADI-P powder diffractometer, (Stoe^®^, Darmstadt, Germany) in transmission geometry by using CuKα_1_ radiation (λ = 1.54056 Å), selected by a curved monochromator Ge (111), with a tube voltage of 40 kV and a current of 40 mA. For LASSBio-1834 (**3**) the measurement was performed in the angular range from 4° to 82.74° (2θ) with steps of 0.015° and integration time of 200 s and for the LASSBio-1835 (**4**), the measurement was performed in the angular range from 8° to 86.74° (2θ) with steps of 0.015° and integration time of 200 s.

### 3.5. Indexing

XRPD data of LASSBio-1834 (**3**) and LASSBio-1835 (**4**) were used to index the first 26 reflections of the patterns using the TOPAS-Academic v.6 software program [19], and Pawley fits [20] were performed, thus resulting in a monoclinic crystal system with space group *P*2_1_/*c*.

### 3.6. Structure Determination and Rietveld Refinement

To determine the crystal structure of both compounds we used the XRPD data, the information previously found in the indexing step and the 3D sketch of LASSBio-1834 (**3**) and LASSBio-1835 (**4**), created with the program MarvinSketch version 18.10.0-8214, 2018, ChemAxon (http://www.chemaxon.com). This step was performed using a simulated annealing algorithm implemented into the DASH software program [21], on the basis of previous procedures [22,23,24]. The best result was then considered in the Rietveld refinement of each structure.

In the Rietveld refinement the background was fit using a 19-term Chebyshev polynomial, the peak asymmetry was fit by the simple axial divergence model of Cheary and Coelho [25,26] and the peak profiles were modeled by the Double-Voigt approach [27] with anisotropic peak profiles adjusted using a 4-term spherical harmonics [28,29,30]. Using the Mercury software program [31], we introduced the hydrogen atoms into calculated positions considering the orbital geometry, and the isotropic atomic displacements (*B_iso_*) were constrained to be equal for all non-hydrogen atoms. For hydrogen atoms, the values were 1.2 times the ones referring to the atoms to which they are connected. Their factional coordinates were refined restraining the distances between H atoms to the ones they are associated to.

### 3.7. Molecular Modeling

Potential energy surfaces (PES) between 0° and 180° with a 30° step were calculated with Spartan’16 (Wavefunction, Inc.) with the CAM-B3LYP density functional [32] using the 6-31G(d) basis set. An NBO (Natural Bond Orbitals) [33] analysis was performed with the Gaussian’09 [34] at the CAM-B3LYP/6-311G(d) level, using the available crystallographic coordinates of the evaluated compounds.

### 3.8. Scanning Electron Microscopy

The micrographs were obtained at room temperature and high vacuum conditions. The images were acquired using scanning electron microscope (SEM) equipment JEOL^®^ (Tokyo, Japan), model JCM-6000, operating at 10 kV. The samples were mounted on the stubs using carbon tapes and coated with gold in order to obtain a good contact and to conduct the electrons throughout the samples.

## 4. Conclusions

Using different techniques of structural and morphological analysis, associated to spectroscopic and spectrometric methods and molecular modeling studies, we clearly indicated the presence of a preferred synperiplanar conformation in bioactive 2-thienyl-*N*-acylhydrazone derivatives LASSBio-1834 (**3**) and LASSBio-1835 (**4**), which is maintained by an intramolecular 1,5-N∙∙∙S σ-hole interaction. Moreover, the results obtained from in vitro evaluation confirmed the anticipated multitarget profile of LASSBio-1835 (**4**), which was able to modulate both targets, PDE4 and A2A receptors, with similar potencies. For this reason, it may be considered a promising prototype for the treatment of pulmonary arterial hypertension.

## Figures and Tables

**Table 1:** PDE4 inhibitory activity evaluation of LASSBio-1834 (**3**) and LASSBio-1835 (**4**).

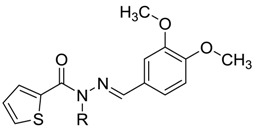				
Compounds	PDE4A1A ^a^	PDE4B1 ^a^	PDE4C ^a^	PDE4D3 ^a^
R = H (**3**)	53%	48%	12%	19%
R = CH_3_ (**4**)	78%	44%	22%	40%

^a^ Percent inhibition determined at a concentration of 10 µM.

## Supplementary Materials

The following are available online at http://www.mdpi.com/1424-8247/11/4/119/s1. Details from Rietveld refinements of the crystal structures; the formation of the crystalline aggregates; DSC curves; ^1^H and ^13^C NMR data; and the chromatograms of (**3**) and (**4**) are available in the Supplementary Materials.
