# Supplementary material for: Design, Synthesis, Experimental and Theoretical Characterization of a New Multitarget 2-Thienyl-N-Acylhydrazone Derivative
Source: Pharmaceuticals (Basel). 2018 Nov 1;11(4):119. doi: 10.3390/ph11040119 (PMC6316713; doi:10.3390/ph11040119)
Supplement: Supplementary file 1 [file pharmaceuticals-11-00119-s001.pdf]

SUPPORTING INFORMATION

Design, synthesis, experimental and theoretical characterization of a new multitarget 2-thienyl-*N*-acylhydrazone derivative

Isadora T. S. Bastos[1], Pedro de Sena M. Pinheiro[2,3], Fanny N. Costa[4], Miguel D. Rocha[2], Carlos Mauricio R. de Sant'Anna[2,5], Delson Braz[6], Everton T. Souza[3,7], Marco A. Martins[3,7], Eliezer J. Barreiro[2,3], Fabio F. Ferreira[4], Regina C. Barroso[1]* and Carlos A. M. Fraga[2,3]*

Table S1. Details from Rietveld refinements of the crystal structure of LASSBio-1834 (3) and LASSBio-1835 (4).

Chemical formula	$C_{14}H_{14}N_2O_3S$	$C_{15}H_{16}N_2O_3S$
Formula weight (g mol^{-1})	290.34	304.36
Crystal system	Monoclinic	Monoclinic
Space group	$P2_1/c$ (Nr. 14)	$P2_1/c$ (Nr. 14)
a, *b*, *c* (Å)	10.18727(19); 16.2201(3);8.34362(14)	11.27114(17); 9.73916(13); 13.6237(2)
β (°)	90.3514(9)	90.4227(8)
Volume (Å^3)	1378.66(4)	1495.45(4)
Z, *Z´*	4, 1	4, 1
ρ_{calc} (g cm^{-3})	1.399	1.352
T (K)	298(2)	298(2)
Data collection		
Diffractometer	STADI P	STADI P
Monochromator	Ge(111)	Ge(111)
Wavelength (Å)	1.54056	1.54056
2θ range (°)	4-82.735	8-86.735
Step size (°)	1.05	1.05
Time per step (s)	200	200
Refinement		
Number of data points	3360	5250
Number of contributing reflections	922	1117
Number of restraints	47	47
Number of refined parameters	96	96
R_p (%)	2.256	2.615
R_{exp} (%)	2.116	2.243
R_{wp} (%)	3.155	3.433
R_{Bragg} (%)	1.022	1.181
χ^2	1.491	1.530

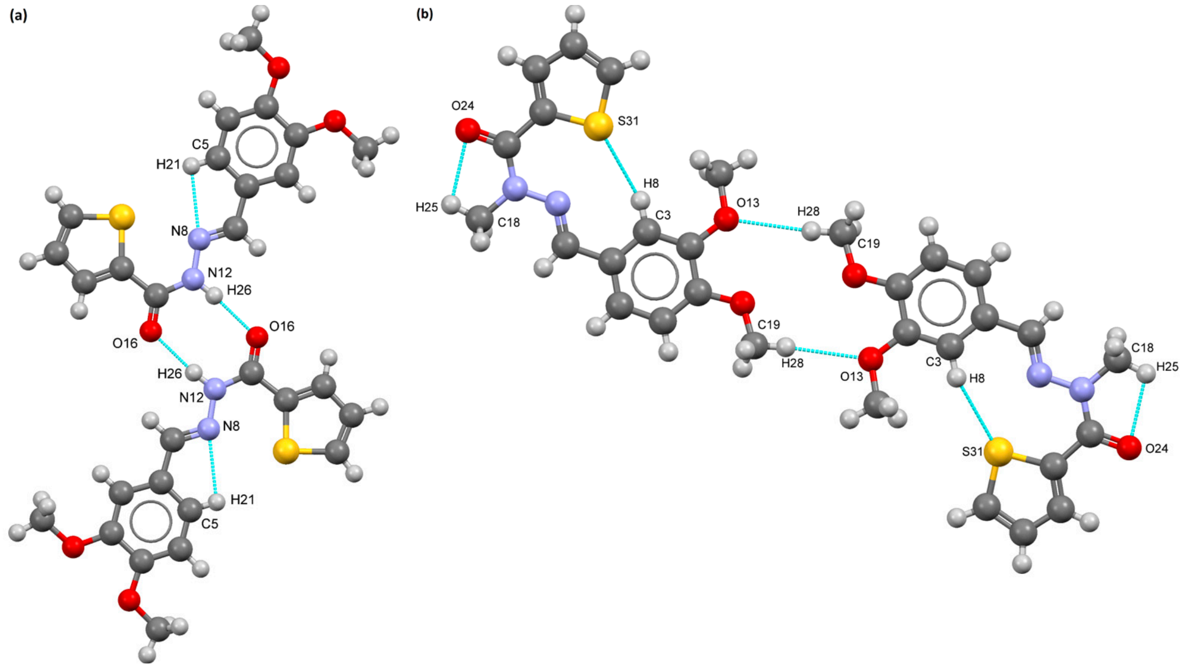

Figure S1. The formation of the crystalline aggregate of LASSBio-1834 (3) molecules (a) and LASSBio-1835 (4) (b) the inter- and intramolecular H-bonds (cyan dashed lines).

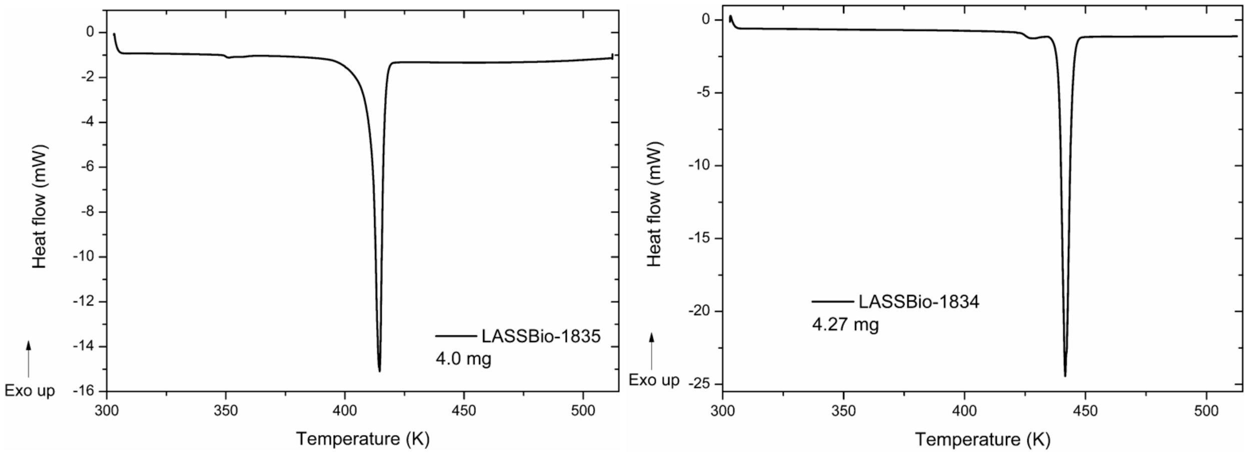

Figure S2. DSC curves of LASSBio-1834 (3) and LASSBio-1835 (4).

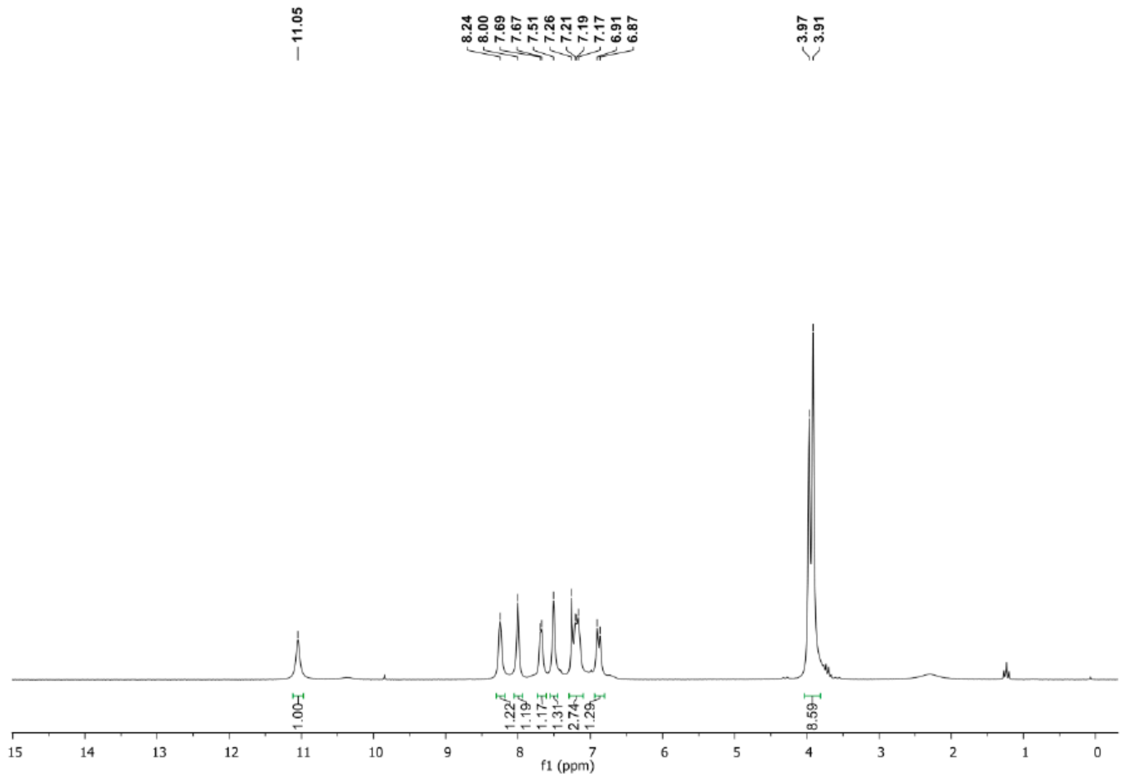

Figure S3. LASSBio-1834 (3) (^{1}H NMR, 200 MHz, $CDCl_3$).

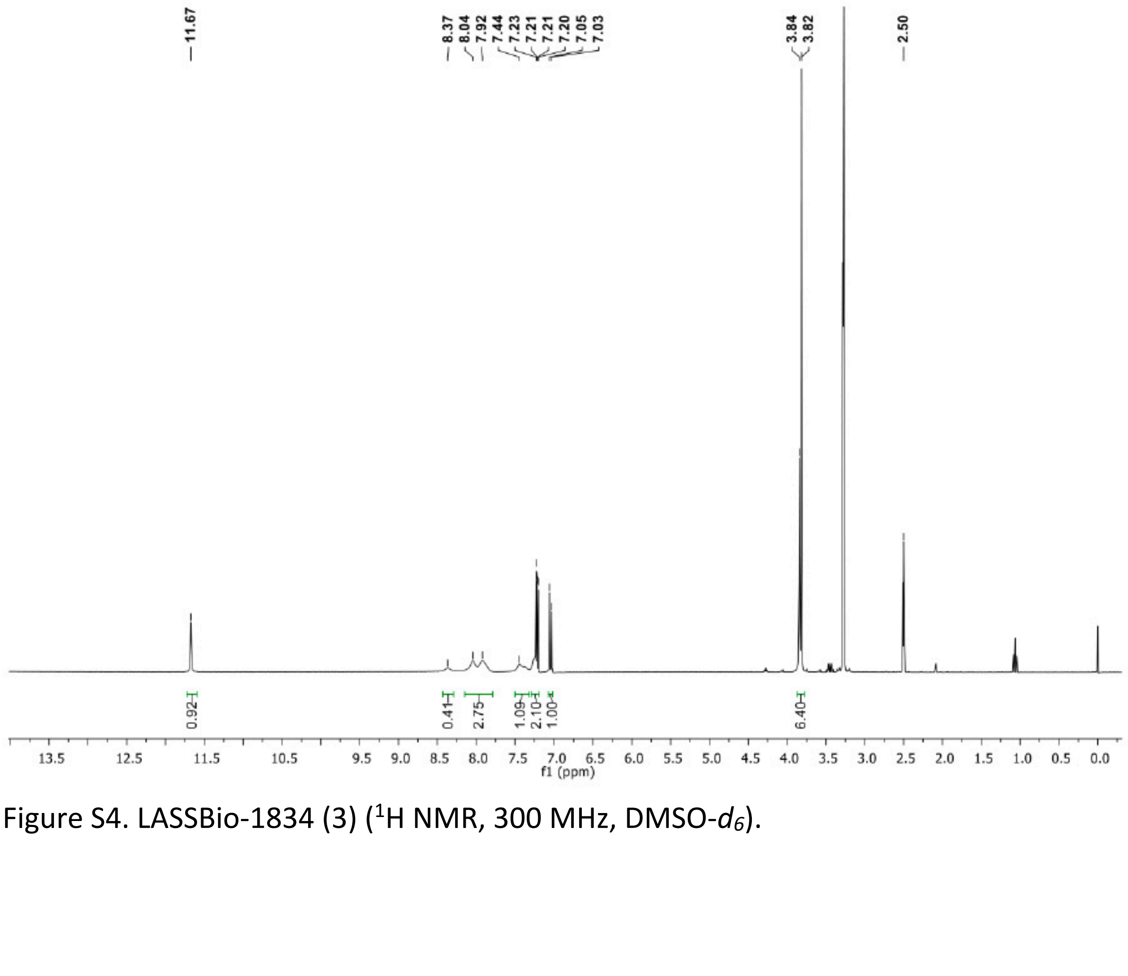

Figure S4. LASSBio-1834 (3) (^{1}H NMR, 300 MHz, DMSO-d_6).

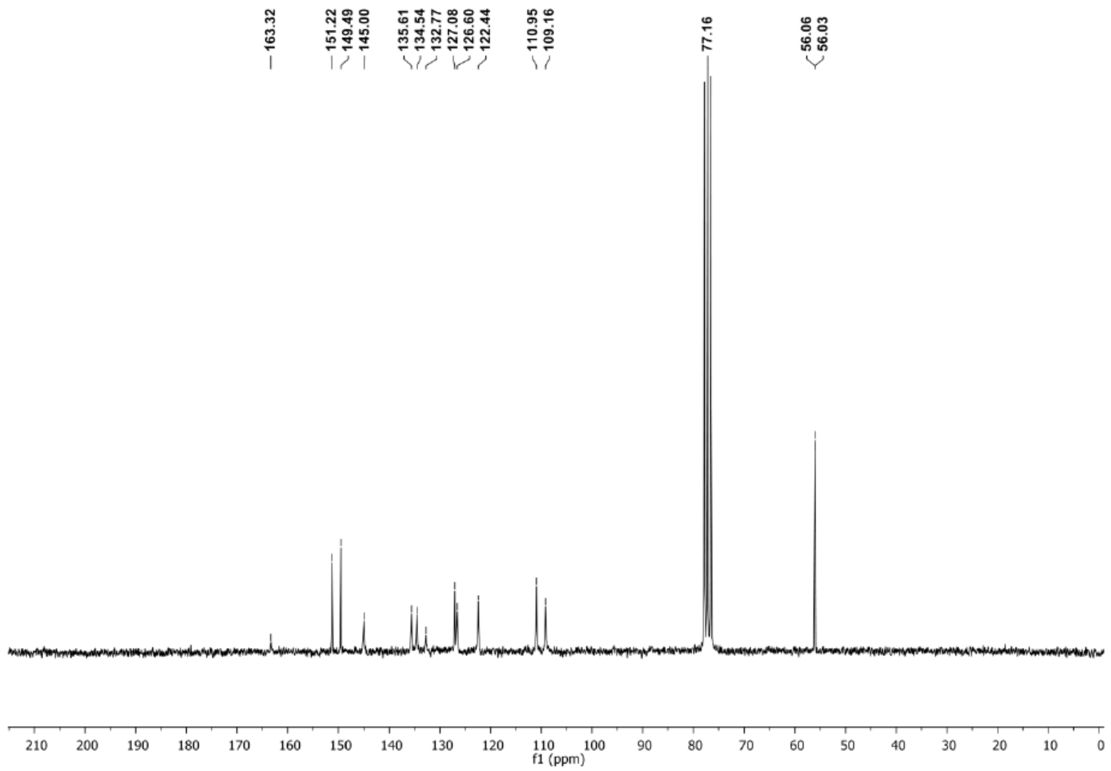

Figure S5. LASSBio-1834 (3) (^{13}C NMR, 50 MHz, $CDCl_3$).

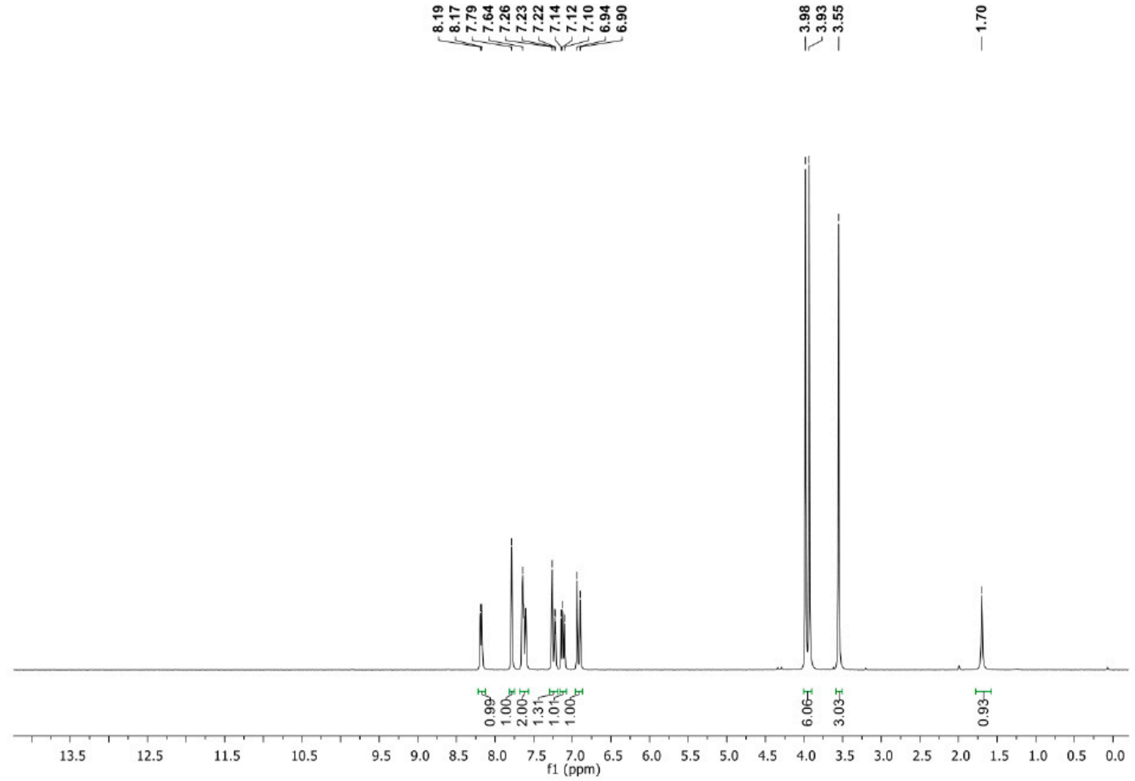

Figure S6. LASSBio-1835 (4) (^{1}H NMR, 200 MHz, $CDCl_3$).

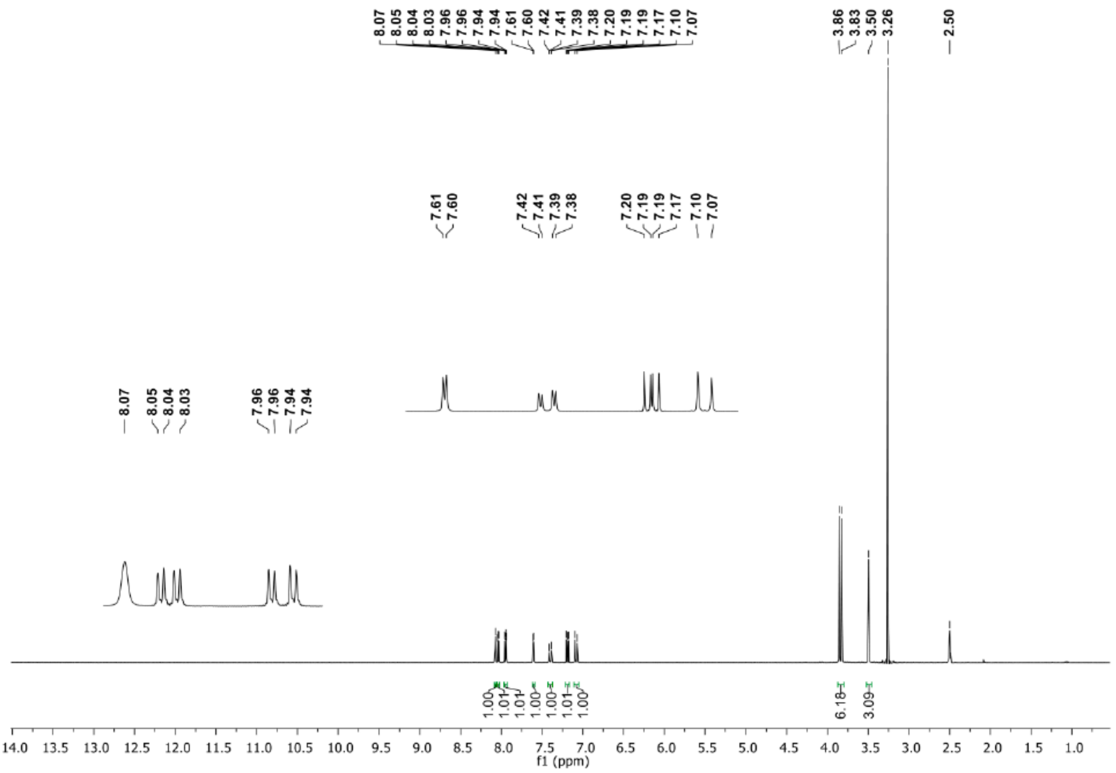

Figure S7. LASSBio-1835 (4) (^{1}H NMR, 300 MHz, DMSO-d_6).

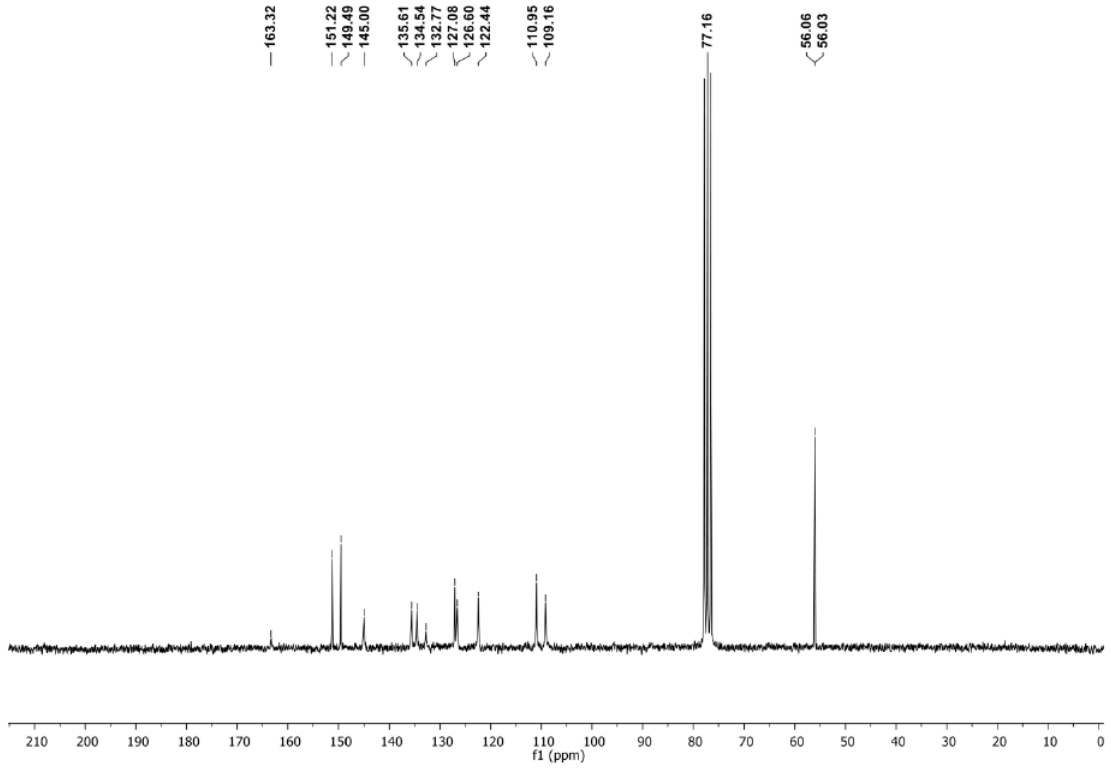

Figure S8. LASSBio-1835 (4) (^{13}C NMR, 50 MHz, $CDCl_3$).

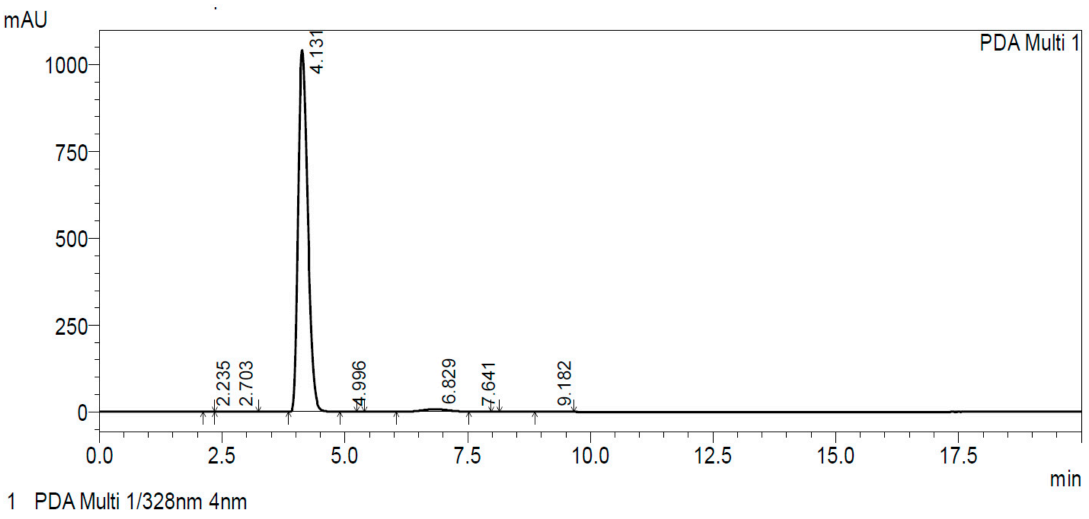

PeakTable

PDA Ch1 328nm 4nm

Peak#	Ret. Time	Area	Height	Area %	Height %
1	2.235	2674	392	0.018	0.037
2	2.703	15659	558	0.108	0.053
3	4.131	14185285	1040120	97.660	99.057
4	4.996	2740	247	0.019	0.024
5	6.829	294821	7366	2.030	0.702
6	7.641	3663	248	0.025	0.024
7	9.182	20299	1093	0.140	0.104
Total		14525142	1050024	100.000	100.000

Figure S9. LASSBio-1834 (3) chromatogram.

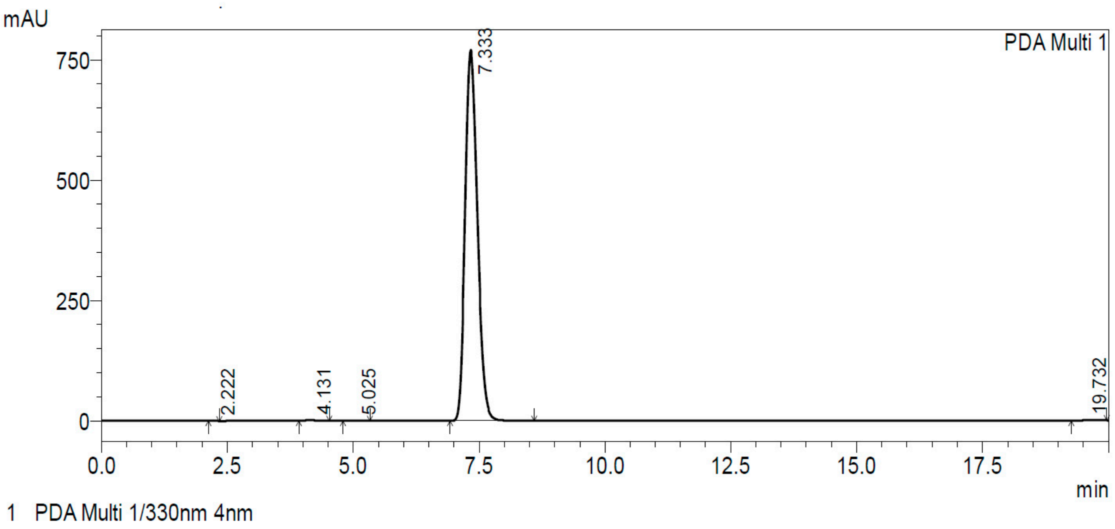

PeakTable

PDA Ch1 330nm 4nm

Peak#	Ret. Time	Area	Height	Area %	Height %
1	2.222	2829	420	0.022	0.054
2	4.131	22991	1656	0.175	0.214
3	5.025	6525	506	0.050	0.065
4	7.333	13068742	769535	99.532	99.493
5	19.732	29152	1339	0.222	0.173
Total		13130239	773456	100.000	100.000

Figure S10. LASSBio-1835 (4) chromatogram.
